# Immunomodulation by Mosquito Salivary Protein AgSAP Contributes to Early Host Infection by *Plasmodium*

**DOI:** 10.1128/mBio.03091-21

**Published:** 2021-12-14

**Authors:** Gunjan Arora, Andaleeb Sajid, Yu-Min Chuang, Yuemei Dong, Akash Gupta, Kristen Gambardella, Kathleen DePonte, Lionel Almeras, George Dimopolous, Erol Fikrig

**Affiliations:** a Section of Infectious Diseases, Department of Internal Medicine, Yale University School of Medicine, New Haven, Connecticut, USA; b Department of Molecular Microbiology and Immunology, Bloomberg School of Public Health, Johns Hopkins University, Baltimore, Maryland, USA; c Unité de Parasitologie et Entomologie, Département de Microbiologie et Maladies Infectieuses, Institut de Recherche Biomédicale des Armées, Marseille, France; d Aix Marseille Université, IRD, AP-HM, SSA, UMR Vecteurs-Infections Tropicales et Méditerranéennes (VITROME), IHU-Méditerranée Infection, Marseille, France; NIAID/NIH

**Keywords:** *Anopheles*, mosquito, *Plasmodium falciparum*, immune regulation, malaria

## Abstract

Malaria is caused when *Plasmodium* sporozoites are injected along with saliva by an anopheline mosquito into the dermis of a vertebrate host. Arthropod saliva has pleiotropic effects that can influence local host responses, pathogen transmission, and exacerbation of the disease. A mass spectrometry screen identified mosquito salivary proteins that are associated with *Plasmodium* sporozoites during saliva secretions. In this study, we demonstrate that one of these salivary antigens, Anopheles gambiae sporozoite-associated protein (AgSAP), interacts directly with Plasmodium falciparum and Plasmodium berghei sporozoites. AgSAP binds to heparan sulfate and inhibits local inflammatory responses in the skin. The silencing of *AgSAP* in mosquitoes reduces their ability to effectively transmit sporozoites to mice. Moreover, immunization with AgSAP decreases the *Plasmodium* burden in mice that are bitten by *Plasmodium*-infected mosquitoes. These data suggest that AgSAP facilitates early *Plasmodium* infection in the vertebrate host and serves as a target for the prevention of malaria.

## INTRODUCTION

Malaria is among the oldest and most pernicious human diseases (1, 2). The latest report from the World Health Organization indicates that malaria caused approximately 230 million clinical episodes and over 400,000 deaths last year (3). *Plasmodium*, the causative agent of malaria, is a parasite transmitted by the bite of an infected *Anopheles* mosquito, primarily Anopheles gambiae in Africa (4). *Plasmodium* sporozoites are deposited into the skin of a vertebrate host as the mosquito probes for blood feeding. Once inside, sporozoites spend several hours at the inoculation site, largely hidden from the immune system in the skin (5). The success of *Plasmodium* infection depends on the ability of sporozoites to evade or modulate the local immune response before reaching the liver, where they invade hepatocytes and establish systemic infection (6, 7).

In recent years, it has been shown that some mosquito proteins act as immune modulators and help in mosquito feeding or pathogen transmission (8–12). Our current understanding of the molecular interactions among host, parasite, and mosquito proteins during the early stage of malaria infection remains fragmentary. Identifying interactions between anopheline salivary proteins and the vertebrate host and/or *Plasmodium* and their overall role in the establishment of *Plasmodium* infection is critical for developing new strategies for malaria elimination (13). Recently, a proteomics-based approach allowed us to identify mosquito salivary proteins that interact with *Plasmodium* sporozoites during mosquito salivation (14). Among these salivary proteins, mosquito gamma interferon (IFN-γ)-inducible lysosomal thioreductase (mosGILT) and sporozoite-associated mosquito salivary protein 1 (SAMSP1) were further shown to directly interact with *Plasmodium* sporozoites and modulate their infectivity and transmission (12, 14). In this study, we have studied the role of Anopheles gambiae sporozoite-associated protein (AgSAP), a novel mosquito salivary antigen, in the early stages of *Plasmodium* infection in the vertebrate host.

## RESULTS

### AgSAP is expressed in A. gambiae salivary glands.

**FIG 1:**
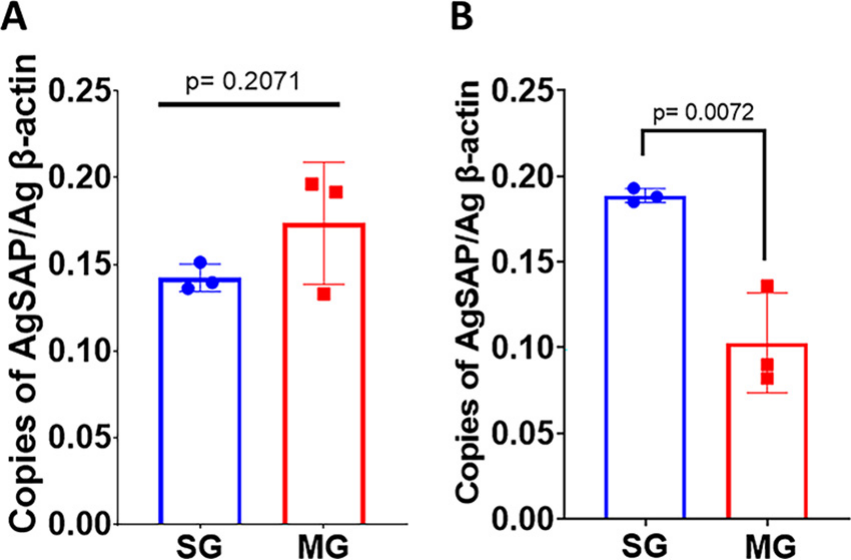
Expression analyses of *AgSAP*. A. gambiae mosquitoes (*n* = 10) (uninfected [A] and infected [21 days postinfection] with P. berghei [B]) were dissected to isolate salivary glands (SGs) and midguts (MGs). The tissues were collected in TRIzol reagent, and total RNA was extracted. qRT-PCR analysis was performed to compare the expression levels of *AgSAP* in SGs and MGs from uninfected mosquitoes (A) and P. berghei-infected mosquitoes (B). The bar graphs represent the relative expression levels of *AgSAP* normalized to the A. gambiae β-actin gene, and error bars represent means ± SD.

Mass spectrometry analysis of sporozoites that were collected from the saliva of *Plasmodium*-infected mosquitoes led to the identification of AGAP004803, which has now been named Anopheles gambiae sporozoite-associated protein (AgSAP). Although AgSAP was present in saliva from uninfected as well as Plasmodium berghei-infected A. gambiae mosquitoes, higher levels were found in the infected fraction (14). Phlyogenetic analyses indicate that AgSAP has homologs in other major *Anopheles* vectors, such as A. stephensi (uncharacterized protein LOC118512319) (61% identity), A. sinensis (54%), A. darlingi (51%), and A. albimanus (51%). The gene is also present in Aedes aegypti, A. albopictus, (45%), and Culex quinquefasciatus (43%) (see Fig. S1 in the supplemental material). In some anopheline species, the AgSAP homolog is named papilin, an extracellular matrix protein present in *Drosophila* that inhibits metalloproteinase protein activity and influences cell rearrangements (15). Expression analyses revealed that *AgSAP* is expressed in both the mosquito salivary gland (SG) and midgut (MG) (Fig. 1A). The expression of *AgSAP* showed an insignificant uptrend in the midgut tissue (Fig. 1A) (*P* = 0.2071). However, upon P. berghei infection, the expression of *AgSAP* was significantly upregulated in the salivary glands compared to its level in the midgut (Fig. 1B) (*P* = 0.0027). This finding suggests that *Plasmodium* parasites induce the expression of AgSAP in a direct or indirect manner.

10.1128/mBio.03091-21.1 FIG S1 Phylogenetic relationships of putative AgSAP homologs. A phylogenetic tree was reconstructed using the maximum likelihood method with the number of bootstraps set to 100 (www.phylogeny.fr). The 'Whelan and Goldman' (WAG) substitution model was selected assuming an estimated proportion of invariant sites (of 0.002) and 4 gamma-distributed rate categories to account for rate heterogeneity across sites. The gamma shape parameter was estimated directly from the data (gamma = 2.742). Reliability for the internal branch was assessed using the approximate likelihood-ratio (aLRT) test (Shimodaira–Hasegawa [SH]-like). Numbers in red represent branch support values. Download FIG S1, TIF file, 0.2 MB. Copyright © 2021 Arora et al. 2021 Arora et al. https://creativecommons.org/licenses/by/4.0/ This content is distributed under the terms of the Creative Commons Attribution 4.0 International license.

### AgSAP binds to the Plasmodium berghei sporozoite surface.

**FIG 2:**
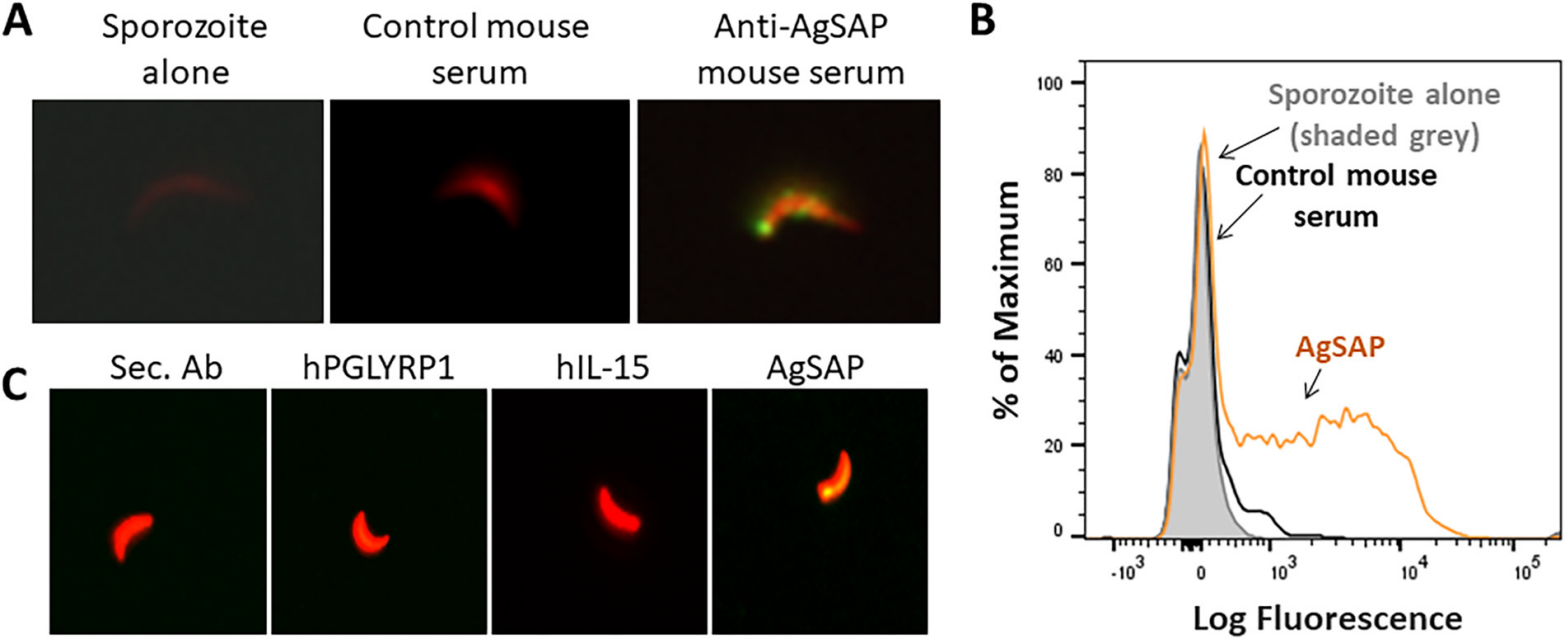
AgSAP binding to P. berghei sporozoites. To examine the interaction of AgSAP with *Plasmodium* sporozoites, P. berghei sporozoites were probed with polyclonal antibodies specific for AgSAP. (A) Sporozoites (red) were labeled with endogenous AgSAP on the surface (green) and probed with goat anti-mouse IgG antibody labeled with the fluorescent dye Alexa Fluor 488. Samples were visualized using a fluorescence microscope at a ×20 magnification. As a control, sporozoites were incubated with serum from naive mice or with secondary anti-mouse IgG antibody only. (B) The binding of AgSAP to sporozoites was analyzed by flow cytometry. *Plasmodium* sporozoites were isolated from infected A. gambiae mosquitoes. Sporozoites were incubated with preimmune serum or AgSAP-specific antibodies generated in mice. After binding, sporozoites were fixed and analyzed by flow cytometry. Binding is compared in the overlay histogram. The sample that had sporozoites incubated with AgSAP-specific antiserum is shown by an orange trace, and the sample with sporozoites and secondary anti-mouse IgG antibody alone is shown in a black trace, while the sporozoites alone are shown in solid gray. (C) To investigate AgSAP binding to *Plasmodium* sporozoites, we used recombinant purified protein. Recombinant AgSAP (100 μg/ml) was incubated with *Plasmodium* sporozoites expressing redstar. The concentration was based on our previous work with mosGILT (14). As controls, recombinant human interleukin-15 (hIL-15) or human peptidoglycan recognition protein 1 (hPGLYRP1) was incubated with sporozoites at the same concentrations. Sporozoites (red) were subsequently probed with mouse anti-His tag primary antibodies and anti-mouse IgG AF488-labeled secondary antibody (Sec. Ab) (green).

To examine whether AgSAP interacts with *Plasmodium* sporozoites, we first cloned and purified recombinant AgSAP from an Escherichia coli protein expression system and then generated mouse polyclonal antisera against the recombinant protein. An immunofluorescence assay was used to validate the association of AgSAP with *Plasmodium* sporozoites as suggested by the results of a mass spectrometry screen (14). P. berghei sporozoites were probed with polyclonal antibodies specific for AgSAP together with mouse serum as a negative control (Fig. 2A). Sporozoites had clear staining of native AgSAP at their surface when probed with goat anti-mouse IgG antibody labeled with the fluorescent dye Alexa Fluor 488 (AF488) (green). No signal was detected on sporozoites incubated with serum from naive mice or with secondary anti-mouse IgG antibody only. The binding of AgSAP to sporozoites was also demonstrated by flow cytometry (Fig. 2B). As shown in the overlay histogram, sporozoites incubated with AgSAP-specific antiserum showed binding, compared to secondary anti-mouse IgG antibody alone. It is worth noting that the majority of, but not all, sporozoites have AgSAP present on their surface (secondary antibody control, ∼3.59%; control mouse serum, ∼9.09%; AgSAP, ∼76.2%).

To further examine the binding of AgSAP to P. berghei sporozoites, recombinant AgSAP (rAgSAP) was incubated with sporozoites isolated from salivary glands. As controls, two unrelated proteins, recombinant human interleukin-15 (hIL-15) (6×His tagged) and human peptidoglycan recognition protein 1 (hPGLYRP1) (8×His tagged) were incubated with sporozoites at similar concentrations. Binding was probed with mouse anti-His tag primary antibodies and anti-mouse IgG Alexa Fluor 488-labeled secondary antibody (green). AgSAP was found to bind to sporozoites, as observed using fluorescence microscopy, while no such binding was seen with IL-15 or PGLYRP1 (Fig. 2C). Together, these studies reveal that AgSAP interacts with the surface of *Plasmodium* sporozoites.

### AgSAP is present on the Plasmodium falciparum sporozoite surface.

**FIG 3:**
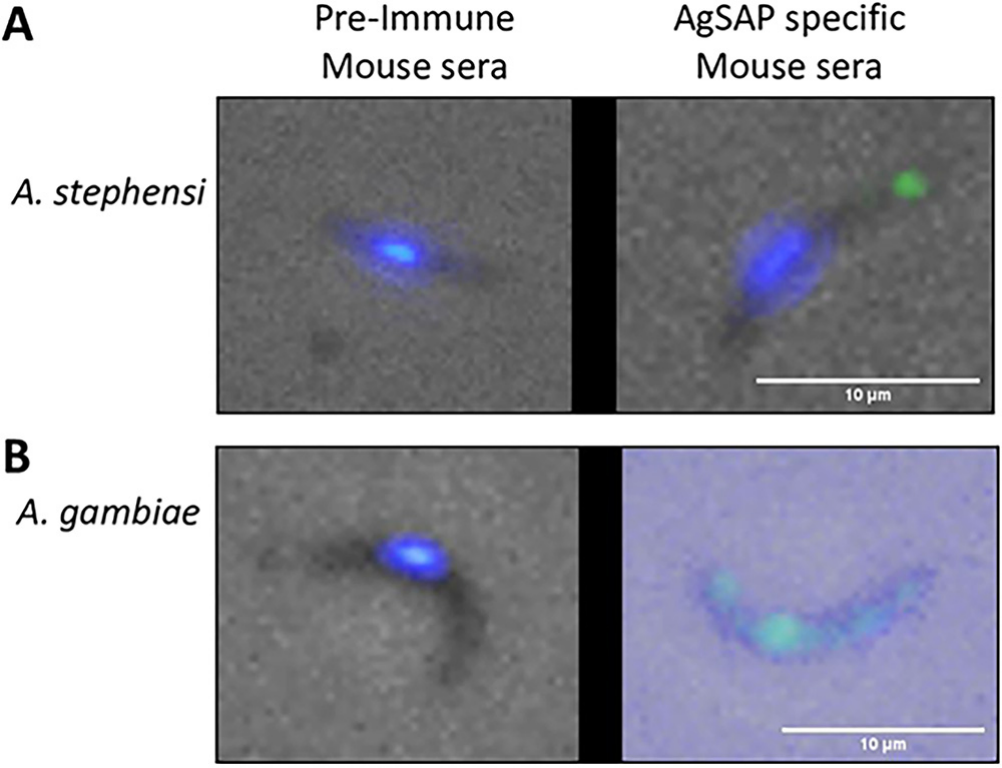
AgSAP binds Plasmodium falciparum sporozoites. P. falciparum sporozoites were probed with polyclonal antibodies specific for AgSAP. *Plasmodium* sporozoites were isolated from A. stephensi (A)- or A. gambiae (B)-infected mosquitoes. AgSAP binding at the surface of the sporozoite was examined by probing with goat anti-mouse IgG antibody labeled with the fluorescent dye Alexa Fluor 488 and visualized under a fluorescence microscope (Evos). As a control, sporozoites were incubated with serum from naive mice or with secondary anti-mouse IgG antibody only.

To examine whether AgSAP interacts with sporozoites of the human malaria parasite Plasmodium falciparum, we performed an immunofluorescence assay (IFA) using polyclonal antibodies specific for AgSAP (Fig. 3A and B). The P. falciparum sporozoites for this experiment were isolated from either A. stephensi (Fig. 3A) or A. gambiae (Fig. 3B) mosquitoes. AgSAP was detected on the surface of P. falciparum sporozoites when probed with goat anti-mouse IgG antibody labeled with the fluorescent dye Alexa Fluor 488. Binding was absent in sporozoites that were incubated with serum from naive mice or with secondary anti-mouse IgG antibody only. These results show that AgSAP is a sporozoite-associated protein that binds to P. falciparum and P. berghei sporozoites.

### AgSAP does not affect the viability of *Plasmodium* sporozoites.

We next examined whether the interaction between sporozoites and AgSAP can influence sporozoite viability, a critical requirement for *Plasmodium* sporozoites to establish infection. *Plasmodium* sporozoites were incubated with AgSAP or bovine serum albumin (BSA) (as a negative control), and viability was measured using the cell-permeant dye calcein-acetoxymethyl (calcein-AM), which becomes fluorescent after hydrolysis by intracellular esterases. As expected, AgSAP incubation did not affect the sporozoites’ viability as visualized under a fluorescence microscope (Fig. S2).

10.1128/mBio.03091-21.2 FIG S2 Effect of AgSAP on the viability of *Plasmodium* sporozoites. *Plasmodium* sporozoites were incubated with either PBS, BSA, or AgSAP for 30 min. Sporozoite viability was assessed using calcein-AM dye, and the sporozoites were visualized under a fluorescence microscope (Evos). Download FIG S2, TIF file, 0.1 MB. Copyright © 2021 Arora et al. 2021 Arora et al. https://creativecommons.org/licenses/by/4.0/ This content is distributed under the terms of the Creative Commons Attribution 4.0 International license.

### AgSAP antibodies are elicited by natural mosquito bites.

**FIG 4:**
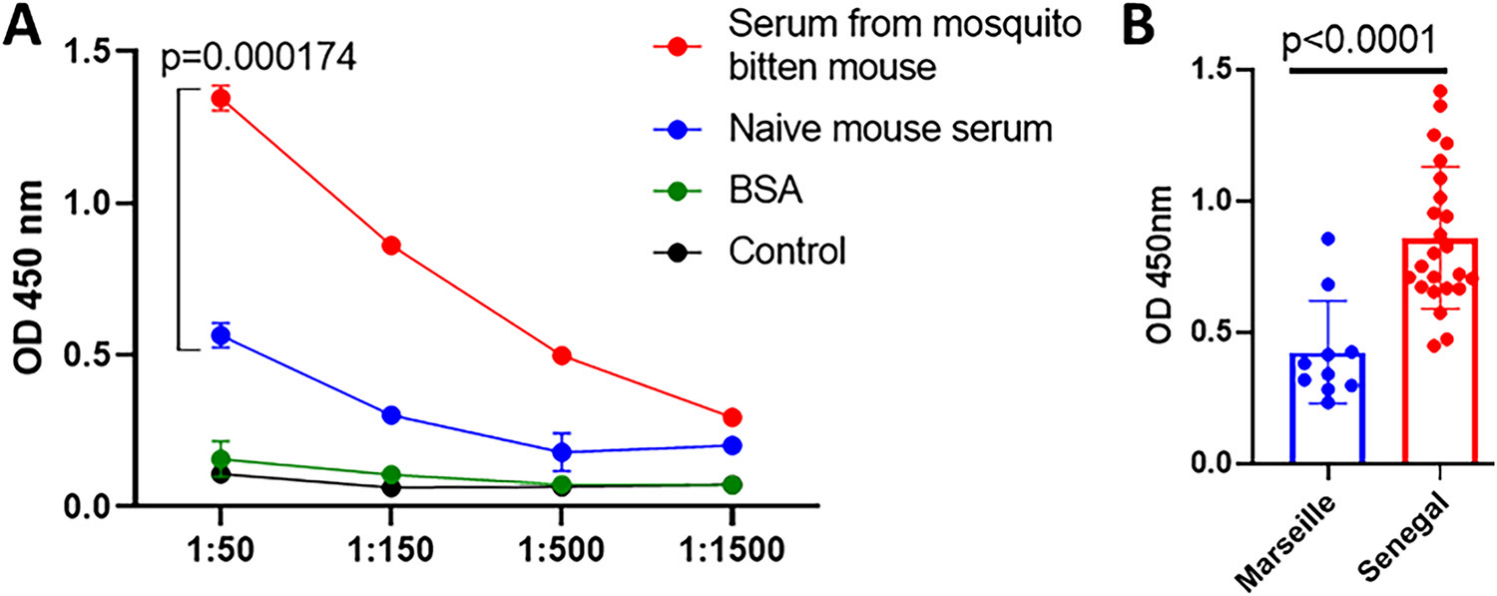
AgSAP-specific antibody responses in mice and humans exposed to *Anopheles* mosquitoes. (A) The AgSAP-specific IgG response was measured in mice bitten by A. gambiae mosquitoes. C57BL/6 mice were bitten by A. gambiae mosquitoes over 6 weeks. IgG antibodies against AgSAP were measured in exposed mice as well as naive mice that were not exposed to *Anopheles* bites. (B) To assess the antibody response in humans, sera from individuals living in an area where malaria is endemic (Senegal) were compared with sera from individuals where malaria is not prevalent (Marseille). OD, optical density.

Since AgSAP affects *Plasmodium* infection in mice, we determined whether AgSAP-specific IgG was produced in mice bitten by A. gambiae mosquitoes. We found that laboratory mice bitten by A. gambiae mosquitoes (exposed to >200 mosquito bites at six exposure times) developed IgG antibodies against AgSAP and also recognized native AgSAP in mosquito saliva (Fig. 4A), indicating that the protein is secreted into the vertebrate host. Furthermore, we assessed the antibody response in humans, which also demonstrated similarities to the murine response. Sera from individuals living in an area where malaria is endemic (Senegal) had higher IgG reactivity to AgSAP than individuals where malaria is absent (Marseille, France) (Fig. 4B). As AgSAP was identified as a sporozoite-associated protein, the humoral response to AgSAP suggests a link between the presence of antibodies to AgSAP and exposure to *Plasmodium* in an area where malaria is endemic.

### *AgSAP* knockdown mosquitoes transmit *Plasmodium* less efficiently to mice.

**FIG 5:**
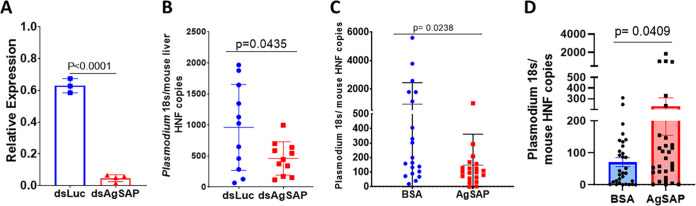
Effect of AgSAP knockdown on *Plasmodium* transmission by *Anopheles* mosquitoes. (A) To study the influence of endogenous AgSAP on *Plasmodium* transmission, double-stranded RNA (dsRNA) against *AgSAP* or the luciferase gene was injected into *Anopheles* mosquitoes infected with P. berghei (13 days postinfection) to decrease the expression of *AgSAP*. Four days after injection of the dsRNA, the expression of *AgSAP* was compared with that of the control dsRNA (ds-luciferase [ds*luc*]) using a qRT-PCR assay. (B) To determine whether a reduction of *AgSAP* has an impact on *Plasmodium* transmission, mice were exposed to three infected mosquitoes. The parasite load was determined in the mouse liver 42 h after mosquito bites. The role of the reduction in the expression of *AgSAP* in *Anopheles* mosquitoes in *Plasmodium* transmission was analyzed by exposing mice to the infected mosquitoes and comparing parasite loads in the mouse livers. (C) In addition, we determined whether immunizing mice with AgSAP would influence mosquito-borne *Plasmodium* infection. Mice immunized with AgSAP or BSA were exposed to infectious mosquito bites, and liver parasite burdens were compared 42 h after mosquito bites. (D) Consistent with this observation, P. berghei sporozoites treated with rAgSAP and injected into mice displayed a significant increase in the liver burden compared to control sporozoites following intradermal injection. The bars in the graphs represent the relative expression levels of *Plasmodium* 18S normalized to the mouse β-actin gene, and error bars represent means ± SD.

To study the influence of endogenous AgSAP on *Plasmodium* transmission, *AgSAP* double-stranded RNA (dsRNA) (ds*AgSAP*) was injected into *Anopheles* mosquitoes infected with P. berghei at 13 days postinfection (dpi) to decrease the expression of *AgSAP*. Four days after injection of the dsRNA, the expression of *AgSAP* was reduced >90% in the ds*AgSAP*-injected mosquitoes compared to the control dsRNA-injected group of mosquitoes (Fig. 5A) (luciferase dsRNA [ds*luc*]). To determine whether a reduction of *AgSAP* has an impact on *Plasmodium* transmission, mice were exposed to infected *AgSAP* knockdown mosquitoes. The silencing of *AgSAP* did not affect the feeding of mosquitoes. The parasite load was determined in the mouse liver 42 h after mosquito bites. After the silencing of *AgSAP*, the bites from infected *Anopheles* mosquitoes caused a 2-fold reduction in the *Plasmodium* burden in the mouse liver as measured by the mean expression level of the 18S *Plasmodium* ribosomal gene (Fig. 5B).

In addition, we determined whether immunizing mice with AgSAP would influence mosquito-mediated *Plasmodium* infection. Mice immunized with AgSAP and exposed to infectious mosquito bites developed a significantly lower liver parasite burden than BSA-immunized mice (Fig. 5C). The antibody levels after immunization were confirmed by an enzyme-linked immunosorbent assay (ELISA) (Fig. S3). To further understand the effect of AgSAP on *Plasmodium* infection, sporozoites were coincubated with AgSAP or BSA and injected intradermally. Compared to the control group, the *Plasmodium* burden in the murine liver was high in the AgSAP group (Fig. 5D). Together, these results further demonstrate that AgSAP plays a role in altering the level of initial *Plasmodium* infection in mice.

10.1128/mBio.03091-21.3 FIG S3 AgSAP-specific antibody responses in mice after active immunization. The immune reactivity of mouse antisera with purified AgSAP or BSA was measured by an ELISA. Proteins were immobilized on microtiter wells and probed with mouse antisera after immunization with either AgSAP or BSA. The values plotted represent the means ± standard errors of the means (SEM) from three replicates from a single experiment. Download FIG S3, TIF file, 0.06 MB. Copyright © 2021 Arora et al. 2021 Arora et al. https://creativecommons.org/licenses/by/4.0/ This content is distributed under the terms of the Creative Commons Attribution 4.0 International license.

### AgSAP binds to heparan sulfate and modulates the cutaneous immune response.

**FIG 6:**
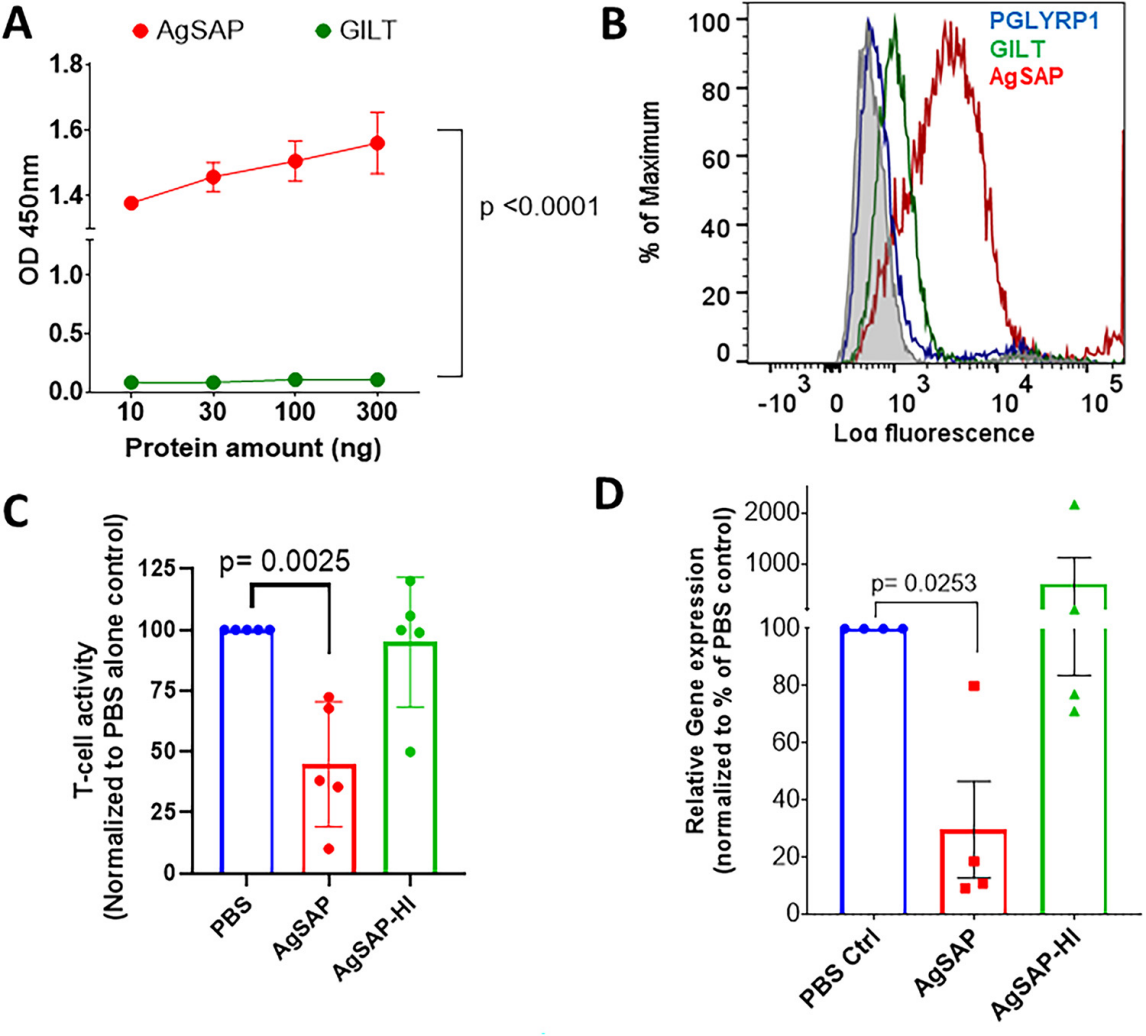
AgSAP interacts with mammalian cell surface proteoglycan and decreases the immune response. AgSAP binds to heparan sulfate, which is present on the host cell surface. (A) In an ELISA, we assessed AgSAP binding to heparan sulfate. As a control, we used mosGILT, another A. gambiae salivary protein present. (B) To investigate the AgSAP interaction with heparan sulfate proteoglycans present on the mammalian cell surface, we used an immortalized T lymphocyte cell line (Jurkat). The overlay histogram shows AgSAP (red) and two other proteins, mosGILT (green) and PGLYRP1 (blue), binding to Jurkat cells. (C) Jurkat Lucia NFAT reporter cells were coincubated with AgSAP or heat-inactivated AgSAP and then stimulated with phorbol myristate acetate-ionomycin. The activity was measured using a CellTiter-Glo assay, and the relative luminescence values were compared. (D) The effect on the Jurkat cell response was also measured at the RNA level by comparing the expression levels of TNF-α.

Heparan sulfate is an acidic polysaccharide belonging to the glycosaminoglycan family, which is present on the mammalian cell surface (16). Once inside the host, *Plasmodium* sporozoites are known to bind heparan sulfate proteoglycans (HSPGs), which activate sporozoites to begin the invasion process (17). Since AgSAP is present on the sporozoite surface, we assessed whether AgSAP has the ability to interact with heparan sulfate. In an ELISA, we show that recombinant AgSAP binds to heparan sulfate (Fig. 6A). As a control, we used mosGILT, another A. gambiae salivary protein present on the surface of sporozoites, which did not bind to heparan sulfate (Fig. 6A).

To investigate if AgSAP can interact with heparan sulfate proteoglycans present on the mammalian cell surface, we used a Jurkat cell line, which is known to have heparan sulfate proteoglycans (18). The Jurkat cell line is an immortalized T lymphocyte cell line that is used to study immune cell signaling, cytokine expression, and interactions between immune receptors and proteoglycans (19–22). AgSAP shows significant binding to Jurkat cells compared to two control proteins, mosGILT (A. gambiae recombinant protein containing an 8×His tag) and PGLYRP1 (Fig. 6B). To assess the effect of AgSAP on immune cells and cytokine production, Jurkat cells were coincubated with AgSAP or heat-inactivated AgSAP (HI-AgSAP) and then stimulated with phorbol myristate acetate (PMA) and ionomycin. PMA-ionomycin activates the protein kinase C and calcium signaling pathways, which results in T cell activation and the expression of proinflammatory cytokines such as tumor necrosis factor alpha (TNF-α) and IFN-γ (23–26). We observed inhibition of T cell activity in the expression of TNF-α and overall Jurkat cell activity in the presence of AgSAP (Fig. 6C and D). In the presence of HI-AgSAP, no such inhibition was observed. We also confirmed that AgSAP does not affect the viability of Jurkat cells (Fig. S4).

10.1128/mBio.03091-21.4 FIG S4 Effect of AgSAP on the viability of Jurkat cells. Jurkat cells were incubated in the absence and presence of different concentrations of AgSAP for 48 h in a 96-well plate, keeping the final volume at 200 μl. Viability was assessed by a CellTiter-Glo assay, and luminescence was measured using a Synergy Mx plate reader (BioTek). The graph shows the effect of different concentrations of AgSAP on Jurkat cells. Results from one independent experiment performed in triplicates are shown here. Download FIG S4, TIF file, 0.08 MB. Copyright © 2021 Arora et al. 2021 Arora et al. https://creativecommons.org/licenses/by/4.0/ This content is distributed under the terms of the Creative Commons Attribution 4.0 International license.

**FIG 7:**
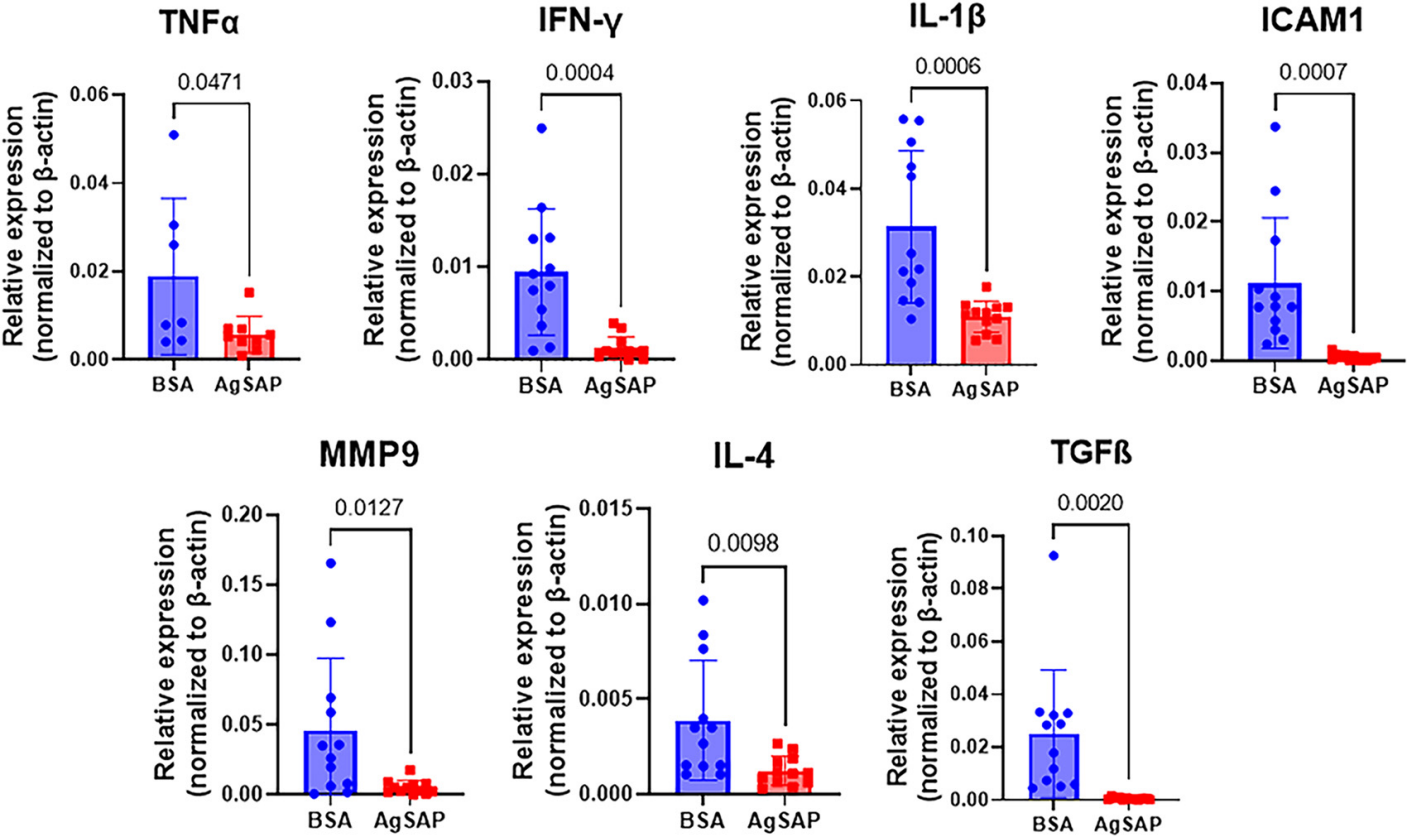
AgSAP inhibits the inflammatory response in mouse skin. The effect of AgSAP on the expression of 7 inflammation-related genes, TNF-α, IL-1β, IFN-γ, IL-4, MMP-9, TGF-β, and ICAM-1, were measured in mouse ears after intradermal injections. BSA was injected into the other ears of the mice. After 6 h, the gene expression levels of TNF-α, IL-1β, IFN-γ, IL-4, MMP-9, TGF-β, and ICAM-1 were compared in both groups by qRT-PCR.

The interaction of AgSAP with heparan sulfate may also influence inflammation since heparan sulfate participates in the recruitment of leukocytes to the site of inflammation and the release of proinflammatory cytokines such as TNF-α, IL-1β, and IL-6 (27, 28). In response to *Plasmodium* sporozoites, host cells produce TNF-α, which can directly or indirectly inhibit the ability of sporozoites to establish *Plasmodium* infection (29). To further understand the effect of AgSAP on the immune response at the inoculation site, AgSAP was intradermally injected into the mouse ear. As a control, BSA was injected into the other ear of the same mice. After 6 h, the expression levels of various immune effectors, IL-6, TNF-α, IL-1β, IFN-γ, IL-4, matrix metalloproteinase 9 (MMP-9), transforming growth factor β (TGF-β), ICAM-1, IL-10, VCAM1, IL-12a, IL-17, and CCL2, were compared in both groups (Fig. 7 and Fig. S5). These cytokines, chemokines, and other immune effectors were selected based on their role in inflammatory responses in the skin, during a mosquito bite, or immune responses related to HSPGs (30–35). The intradermal inoculation of AgSAP into the mouse ear decreased the expression of TNF-α, IL-1β, IFN-γ, IL-4, MMP-9, TGF-β, and ICAM-1 compared to the BSA control (Fig. 7 and Fig. S5). Overall, these results indicate that AgSAP can modulate host immune responses at the site of inoculation.

10.1128/mBio.03091-21.5 FIG S5 AgSAP inhibits the inflammatory response in mouse skin. The effects of AgSAP on the expression of six inflammation-related genes, IL-6, IL-10, VCAM1, IL-12a, IL-17, and CCL2, were measured after intradermal injections into mouse ears. BSA was injected into the other ears of the same mice. Download FIG S5, TIF file, 0.1 MB. Copyright © 2021 Arora et al. 2021 Arora et al. https://creativecommons.org/licenses/by/4.0/ This content is distributed under the terms of the Creative Commons Attribution 4.0 International license.

## DISCUSSION

In arthropod-transmitted diseases, the role of vector salivary proteins has garnered interest over the last decade. The primary purpose of arthropod saliva is to facilitate blood feeding, and saliva can also affect pathogen transmission, either by modulating the host microenvironment at the bite site or by directly interacting with the pathogen. Mosquito saliva contains hundreds of proteins, many of them possessing antiautophagy, antihemostatic, anti-inflammatory, or immunomodulatory properties. Several studies have demonstrated that mosquito saliva can facilitate the transmission of viruses and other pathogens. The passive transfer of antibodies against salivary gland extract (SGE) results in a lower *Plasmodium* burden in mice. AgTRIO and SAMSP1 are two protein candidates that may play important roles in pathogen transmission, while other salivary proteins, such as GILT, inhibit *Plasmodium* infection. Many other mosquito salivary proteins, including gSG6, AgBR1, and Nest1, may be involved in hemostasis and immunomodulation, but their role in *Plasmodium* transmission is not clear (8, 11, 36). The role of salivary protein is not restricted to these antigens, and the role of additional targets such as AeSNAP, a calcium transporter ATPase, has also been shown to be important for the transmission of other pathogens such as dengue virus (37). In another recently reported example, a salivary protein, Aedes aegypti venom allergen 1 (AaVA-1), promotes flavivirus transmission by activating autophagy in host immune cells (9). All these studies indicate that mosquito proteins have important pharmacological activities, and the underlying mechanisms of salivary proteins in pathogen transmission should be a priority in vector biology research.

In malaria, recent studies have shown the critical role of different mosquito salivary proteins, including AgTRIO, SAMSP1, and mosGILT, in affecting *Plasmodium* transmission and the overall disease burden in the host (12, 14, 38). Our previous studies have shown specific salivary proteins, such as SAMSP1 and mosGILT, to be associated with the sporozoites within the mosquito. In the present study, we studied the role of AgSAP, another novel sporozoite-associated mosquito salivary protein. We provide evidence that AgSAP interacts with sporozoites, and more interestingly, it can influence host-pathogen interactions and *Plasmodium* infection during the early stage of infection. *AgSAP* silencing in mosquitoes decreases the overall *Plasmodium* burden in the host liver following mosquito-borne infection of mice. Furthermore, cellular assays showed that AgSAP can alter inflammation in the host. We hypothesize that AgSAP binds to sporozoites and manipulates *Plasmodium* infection in the skin by modulating host immune responses.

AgSAP interacts with heparan sulfate proteoglycan (HSPG), which is a linear polysaccharide present on the mammalian cell surface and is a key regulator of the inflammatory response (16). This led us to hypothesize that AgSAP may impact the proinflammatory response, including TNF-α, which is one of the most important soluble effectors against *Plasmodium* infection. In the preerythrocytic stages, TNF-α directly inhibits the ability of *Plasmodium* sporozoites to infect human and rodent hosts (39). This hypothesis is based on the previous observation that mosquito bites decrease TNF-α levels in the serum of humanized mice (30). Upon tissue injury, heparan sulfate fragments are released and promote the release of TNF-α and other proinflammatory cytokines (40, 41). Our data suggest that AgSAP can interact with heparan sulfate and inhibit TNF-α expression in host skin as well as in Jurkat cells, a human T cell line. Furthermore, we also see decreases in the expression levels of IL-1β, IFN-γ, IL-4, MMP-9, TGF-β, and ICAM-1. Overall, our data suggest that AgSAP binds to heparan sulfate and affects proinflammatory responses at the inoculation site.

To understand the effect of AgSAP inhibition on *Plasmodium* infection, we employed different strategies. RNA interference (RNAi)-mediated silencing of *AgSAP* expression in mosquitoes results in a 2-fold reduction of the *Plasmodium* burden in the mouse liver. When AgSAP-immunized mice were challenged with *Plasmodium*-infected mosquitoes, there was a modest decrease in the *Plasmodium* burden in the liver. Administering AgSAP antiserum to the mice also led to a similar decrease in the parasite burden. Finally, the *Plasmodium* burden in the liver was found to be higher in mice that received sporozoites mixed with AgSAP protein. Overall, these results further confirmed that AgSAP might play an important role in the preerythrocytic stages of *Plasmodium* infection.

After continuous exposure to mosquito bites, humans develop a humoral response against mosquito salivary proteins, and this can be useful for surveillance and as a biomarker for exposure to bites and malaria disease risk (42). Individuals living in an area where malaria is endemic develop a humoral immune response to some salivary antigens such as AgSAP, SAMSP1, SG6, and 5-nucleotidase but not to other proteins like AgTRIO (12, 38, 43). Future studies are needed to understand if combining several salivary proteins will be a better approach than using individual proteins for identifying exposure to mosquitoes and malaria disease risk in areas of endemicity.

In conclusion, we have assessed the role of the novel salivary antigen AgSAP in the initial stages of *Plasmodium* infection and immunomodulation of the host response. Understanding the diverse role of salivary antigens in the early events associated with sporozoite infection of the vertebrate host will allow the development of mosquito antigen-based vaccines and other strategies for malaria intervention.

## MATERIALS AND METHODS

### Ethics statement.

All mice were housed by the Yale Animal Resource Center at Yale University and handled according to the NIH *Guide for the Care and Use of Laboratory Animals* (44). The experiments designed for these studies were approved by the Institutional Animal Care and Use Committee of Yale University (protocol number 2020-07941). Human plasma was collected after institutional review board approval. The Senegal National Ethics Committee (Senegal) and the Marseille-2 Ethical Committee (France) approved the collection of human serum samples (number 2006-A00581-50). Detailed information about these samples was provided previously (38, 45).

### Animals and mosquitoes.

Anopheles gambiae (4ARR strain; MRA-121) mosquitoes were raised at 27°C with 80% humidity under a 12-h/12-h light/dark cycle and maintained with 10% sucrose. A. gambiae (Keele strain) and A. stephensi (Liston strain) were raised at the insectary of the Johns Hopkins Malaria Research Institute (JHMRI) under the same rearing conditions. Swiss Webster and C57BL/6 mice (5- to 6-week-old females) were purchased from Charles River Laboratories (Wilmington, MA).

### Plasmodium berghei infection.

P. berghei (NK65 redstar) infection was maintained in 6- to 8-week-old female Swiss Webster mice as described previously (14, 46). Briefly, P. berghei (NK65 redstar)-infected erythrocytes (RBCs) were inoculated by intraperitoneal injection. Mosquitoes were starved for 20 h and then fed on anesthetized mice with parasitemia levels of >2% (2 to 3 mice/cage of 200 mosquitoes). Sixteen to eighteen days after feeding, mosquito salivary glands (SGs) were observed under a fluorescence microscope to confirm P. berghei infection.

### Plasmodium falciparum infection.

For producing P. falciparum sporozoites, female A. gambiae (Keele strain) or A. stephensi (Liston strain) mosquitoes were fed through an artificial glass membrane feeder with a blood mix containing P. falciparum NF54 (catalog number MRA-1000; BEI Resources) gametocytes adjusted to a final gametocytemia of 0.3% as described previously (14). Sporozoites were isolated from P. falciparum-infected A. gambiae or A. stephensi mosquitoes, and cryopreserved sporozoites were shipped on dry ice from JHMRI to Yale University for AgSAP binding studies.

### Gene cloning and protein purification.

The AgSAP sequence (AGAP004803-PA) was codon optimized and synthesized by IDT and cloned into the E. coli pET28a expression vector (Novagen) using NdeI and HindIII restriction sites, according to the manufacturer’s protocol. AgSAP was also cloned into a pEZT mammalian expression vector (47) using AgeI and NotI restriction sites. The primers utilized for these cloning procedures are listed in Table S1 in the supplemental material. The gene sequence was confirmed by DNA sequencing (Yale University W. M. Keck DNA Sequencing Facility). The pEZT-AgSAP construct was transfected into Expi293F cells according to the manufacturer’s protocol (Thermo Fisher Scientific). The transfected cells expressing A. gambiae AgSAP were maintained at 37°C in Expi293 medium containing a 1% antibiotic-antimycotic solution (Thermo Fisher Scientific). mosGILT and PGLYRP1 were purified as described previously (14, 47).

10.1128/mBio.03091-21.6 TABLE S1 List of oligonucleotides used in this study The oligonucleotides used in this study are detailed. F, forward primer; R, reverse primer. Download Table S1, DOCX file, 0.01 MB. Copyright © 2021 Arora et al. 2021 Arora et al. https://creativecommons.org/licenses/by/4.0/ This content is distributed under the terms of the Creative Commons Attribution 4.0 International license.

AgSAP was also subcloned into an E. coli pGEX-6P2 expression vector (GE Healthcare) using BamHI and NotI restriction sites, and the sequence was confirmed. AgSAP-pET28a or AgSAP-pGEX-6P2 plasmids were transformed into ClearColi BL21 competent cells (InvivoGen). ClearColi cells express modified lipopolysaccharide (LPS), which is less immunogenic to mammalian cells. AgSAP-6×His fusion protein expression was induced with 1 mM isopropyl-β-d-thiogalactopyranoside (IPTG) at 16°C for 12 to 16 h. The cells were sonicated in lysis buffer (10 mM Tris-Cl [pH 8.0], 300 mM NaCl, 5 mM β-mercaptoethanol, complete EDTA-free protease inhibitor tablets [Sigma-Roche], and 1 mM phenylmethylsulfonyl fluoride [PMSF]). Soluble 6×His-AgSAP was purified from the cytosolic fraction using nickel-nitrilotriacetic acid (NTA) agarose resin according to the manufacturer’s protocol (Qiagen). Glutathione *S*-transferase (GST)–AgSAP protein expression was induced with 1 mM IPTG at 37°C for 3 to 4 h. The cells were sonicated in lysis buffer (10 mM Tris-Cl [pH 8.0], 300 mM NaCl, 1 mM dithiothreitol [DTT], complete EDTA-free protease inhibitor tablets [Sigma-Roche], and 1 mM PMSF). Soluble GST-AgSAP was purified from the cytosolic fraction using GST-Sepharose resin according to the manufacturer’s protocol (Sigma). Purified His-AgSAP and GST-AgSAP were concentrated with a 3-kDa-cutoff Amicon filter (EMD Millipore), and buffer was exchanged three times with phosphate-buffered saline (PBS).

### Salivary gland and sporozoite isolation.

SGs were dissected from mosquitoes under a microscope and resuspended in PBS or RPMI 1640 medium. Sporozoites were isolated from the SGs by repeated passaging through a 28 1/2-gauge insulin syringe. The sporozoite SG mixture was then centrifuged at a low speed (1,500 rpm) for 5 min to remove debris. The supernatant was centrifuged at 17,200 × *g* for 10 min, the sporozoite pellet was resuspended in PBS or RPMI 1640 medium, and the sporozoites were counted using a hemocytometer.

### Immunofluorescence assay.

Isolated sporozoites were incubated with either AgSAP-specific polyclonal antibodies or prebleed control mouse sera at 4°C for 1 h. The sporozoites were washed with PBS twice, and the supernatant was discarded to remove unbound antibodies. The sporozoites were subsequently fixed with 2% paraformaldehyde for 30 min at room temperature. After the removal of the fixative, sporozoites were incubated with a goat anti-mouse Alexa Fluor 488 secondary antibody (Thermo Fisher Scientific) (1:1,000) for 30 min. The sporozoites were again washed two times with PBS. Sporozoites were viewed using an Evos FL auto cell imaging system (Thermo Fisher Scientific).

For the protein binding assays, isolated sporozoites were incubated with either AgSAP, hPGLYRP1 (47), or hIL-15 at 4°C for 1 h as described previously (12). The sporozoites were washed with PBS twice, and the supernatant was discarded to remove unbound protein. The sporozoites were subsequently fixed with 2% paraformaldehyde for 30 min at room temperature. After the removal of the fixative, sporozoites were incubated with purified anti-His tag antibody generated in mice (BioLegend) (1:500) for 20 min. The excess antibody was washed with PBS, and sporozoites were probed with a goat anti-mouse Alexa Fluor 488 secondary antibody (Thermo Fisher Scientific) (1:1,000) for 30 min. The sporozoites were again washed two times with PBS. Sporozoites were viewed using an Evos FL auto cell imaging system (Thermo Fisher Scientific).

### Mosquito gene expression by qRT-PCR.

Total RNA was extracted from mosquito tissues in TRIzol reagent (Thermo Fisher Scientific), and cDNA was prepared using an iScript kit (Bio-Rad). Quantitative real-time PCR (qRT-PCR) was done on a CFX96 real-time system (Bio-Rad) using iTaq SYBR green supermix (Bio-Rad). PCR involved an initial denaturation step at 95°C for 3 min and 40 cycles of 10 s at 95°C, 10 s at 60°C, and 10 s at 72°C. Fluorescence readings were taken at 72°C after each cycle. The relative expression of *AgSAP* was normalized to A. gambiae actin mRNA using the comparative ΔΔ*C_T_* method.

### Mouse immunization with AgSAP.

C57BL/6 mice (6-week-old females) were immunized once with either 10 μg of AgSAP or BSA emulsified in complete Freund’s adjuvant (Thermo Fisher Scientific), followed by two boosts of the respective antigen with incomplete Freund’s adjuvant (Thermo Fisher Scientific) every 14 days. At the end of the experiment, serum was collected from each mouse, and the titers were tested by an enzyme-linked immunosorbent assay (ELISA) to confirm antigen-specific antibodies for each mouse.

### Enzyme-linked immunosorbent assays.

Microtiter plates (MaxiSorp 96-well plates; Thermo Fisher Scientific) were coated with 100 ng AgSAP or BSA in PBS at 4°C overnight. The wells were washed three times with PBS-T (PBS with 0.1% Tween 20). The wells were blocked with 3% BSA in PBS-T for 2 h at 37°C. Serum samples from individual mice were diluted 1:10,000 in 100 μl of blocking buffer and incubated for 1 h at room temperature. The wells were washed four times with 300 μl of PBS-T. After incubation with mouse antiserum, goat anti-mouse horseradish peroxidase (HRP)-conjugated secondary antibody (Thermo Fisher Scientific) was added to the wells at a 1:2,500 dilution for 1 h. The wells were washed three times with 300 μl of PBS-T. One hundred microliters of SureBlue 1-component TMB substrate (SeraCare) was added to the wells, and the mixture was incubated for 10 min in the dark, followed by the addition of 100 μl of 3,3′,5,5′-Tetramethylbenzidine (TMB) stop solution. The absorbance was recorded at 450 nm using a spectrophotometer.

For studying the humoral response in humans, the plates were coated with AgSAP and blocked with BSA as described above. Human plasma was added at a 1:100 dilution in a buffer containing 3% BSA for 1 h at room temperature. The wells were washed four times with 300 μl of PBS-T. Subsequently, anti-human IgG HRP-conjugated antibody was added at a 1:8,000 dilution.

For heparan sulfate interactions, plates were coated with heparan sulfate (2 μg) as described above. After blocking with 3% BSA and washing, either AgSAP or GILT was added at different concentrations at 4°C overnight. The wells were washed, and a 1:1,000 dilution of anti-6×His tag monoclonal antibody (BioLegend) was incubated for 1 h at room temperature. The wells were washed four times with 300 μl of PBS-T. After primary antibody incubation, goat anti-mouse HRP secondary antibody (Thermo Fisher Scientific) was added to the wells at a 1:2,500 dilution for 1 h. The wells were washed four times with PBS-T, SureBlue 1-component TMB substrate (SeraCare) was added to the wells, and the mixture was incubated for 10 min in the dark, followed by the addition of 100 μl TMB stop solution. The absorbance was recorded at 450 nm using a spectrophotometer.

### Infection studies.

Sporozoites were isolated from infected SGs as described above and counted with a hemocytometer. AgSAP or BSA (500 ng) was added to sporozoites (∼2,500) and administered into the dermis of the murine ear (6- to 8-week-old female C57BL/6 mice) with a 31-gauge 0.3-ml insulin syringe as described previously (14). For *Plasmodium* transmission, 1 day before challenge, P. berghei-infected A. gambiae mosquitoes were screened and separated into cups as described previously (14, 38). Each C57BL/6 mouse immunized with either BSA or AgSAP was exposed to three infected mosquitoes, and mosquitoes were allowed to bite for 30 min. For analysis of the *Plasmodium* burden in the hepatic tissue, murine livers were harvested at 42 h postinfection. The whole liver was homogenized in 4 ml of TRIzol reagent (Thermo Fisher Scientific). Total RNA was purified from 0.5 ml of the homogenate according to the manufacturer’s protocol and resuspended in 500 μl of molecular-biology-grade water. Following the extraction of RNA, cDNA was synthesized from 3 μg of total RNA using an iScript kit (Bio-Rad). qRT-PCR was performed in triplicates by targeting the P. berghei 18S rRNA gene and normalizing gene expression to mouse hepatocyte nuclear factor 1 alpha (*HNF*) (Table S1) by the comparative threshold cycle (*C_T_*) method, as described above.

### Intradermal inoculation of *Plasmodium* sporozoites mixed with AgSAP.

Sporozoites were counted with Incyto C-Chip hemocytometers (Neubauer Improved). Sporozoites were washed three times with RPMI 1640 medium. Washed sporozoites were incubated with AgSAP or BSA (100 μg/ml) for 1 h on ice. Two thousand five hundred sporozoites in a final volume of 10 μl were injected intradermally into the murine ear (6- to 8-week-old female C57BL/6 mice; Charles River Laboratories) using a 31-gauge 0.3-ml insulin syringe (Easy-Touch). For analysis of the *Plasmodium* burden in the hepatic tissue, murine livers were harvested at 42 to 44 h postinfection. The whole liver was homogenized in TRIzol reagent (Thermo Fisher Scientific) as described above, and purified total RNA was resuspended in water. cDNA was synthesized from total RNA using an iScript kit (Bio-Rad), and qRT-PCR was performed for the P. berghei 18S rRNA gene, normalizing gene expression to the mouse *HNF* gene (Table S1) by the comparative *C_T_* method, as described above.

### dsRNA-mediated gene silencing in *Anopheles* mosquitoes.

RNA interference of genes expressed in mosquito SGs was performed as described previously (14). Briefly, double-stranded RNA (dsRNA) targeting either the *AgSAP* gene or an irrelevant luciferase gene (*luc*) from Renilla reniformis was transcribed using gene-specific primers designed with a T7 promoter and the MEGAscript RNAi kit (Thermo Fisher Scientific) (for primers, see Table S1). P. berghei-infected A. gambiae 4ARR mosquitoes were screened for redstar-positive SGs at 13 dpi, and ds*AgSAP* or ds*luc* at 10 mg/ml (1 μg total dsRNA) was injected into the thorax of the mosquitoes using a Nanoject II Auto-Nanoliter injector (Drummond). Mosquitoes were kept for 4 days after injection of the dsRNA. SGs were dissected at 17 dpi (4 days after dsRNA injection) to monitor gene expression in the presence of ds*luc* and ds*AgSAP* by qRT-PCR (Table S1).

### Jurkat reporter assays.

Jurkat-Lucia NFAT cells are derived from the human T lymphocyte-based Jurkat cell line by the stable integration of a nuclear factor of activated T cells (NFAT)-inducible Lucia reporter construct (catalog number jktl-nfat; InvivoGen). Jurkat cells were cultured in RPMI 1640 medium (catalog number 11875-093; Life Technologies) containing 10% fetal bovine serum (catalog number 26140-079; Life Technologies) and 1% penicillin-streptomycin (Life Technologies). The cells were stimulated with PMA-ionomycin in the absence or presence of AgSAP or heat-inactivated AgSAP. Thirty microliters of the cell supernatant was mixed with 50 μl of Quanti-Luc reagent (InvivoGen), and luminescence was measured using a Synergy Mx plate reader (BioTek).

### Jurkat cell viability assays.

Jurkat cells were incubated in the presence or absence of different concentrations of AgSAP for 48 h. At the end of 48 h, cells were mixed with 50 μl CellTiter-Glo reagent (Promega), and luminescence was measured using a Synergy Mx plate reader (BioTek).

### Jurkat cytokine assays.

Jurkat cells were stimulated with PMA-ionomycin in the absence or presence of AgSAP or heat-inactivated AgSAP for 6 h. RNA was isolated using Qiagen RNeasy columns, and cDNA was prepared using an iScript kit. The expression of TNF-α and IFN-γ was calculated based on the ΔΔ*C_T_* method.

### Analysis of local immune responses after intradermal inoculation of AgSAP.

For the analysis of local immune responses after intradermal injection, AgSAP or BSA was intradermally injected into the left or right ear of the same mice. Recombinant proteins (final concentration of 0.5 μg/μl) were injected intradermally into the dorsal ear as described previously, using a Nanoject II Auto-Nanoliter injector (Drummond) (8). The inoculation sites were marked, and 6 h later, 3-mm punch biopsy specimens were taken from the intradermally injected locations. Total RNA was extracted from the ear biopsy specimen using the RNeasy fibrous tissue minikit (Qiagen), and cDNA was generated in a total volume of 30 μl. Target gene expression was normalized to the mouse β-actin gene, and the expression of 13 genes was analyzed according to ΔΔ*C_T_* calculations.

### Primers.

All primers utilized in these studies are listed in Table S1.

### Statistical analysis.

Data from at least three replicates were used to calculate means or medians for graphing purposes. The differences between the groups were examined by unpaired Student’s *t* test, and the data are presented as means with standard deviations (SD). The differences among more than 3 groups were compared using one-way analysis of variance (ANOVA). A *P* value of <0.05 was considered statistically significant. The analysis, graphs, and statistics of all data were performed using Prism v8.0 and above (GraphPad Software).
