# Supplementary figures and images for: Immunomodulation by Mosquito Salivary Protein AgSAP Contributes to Early Host Infection by Plasmodium
Source: mBio. 2021 Dec 14;12(6):e03091-21. doi: 10.1128/mBio.03091-21 (PMC8669493; doi:10.1128/mBio.03091-21)

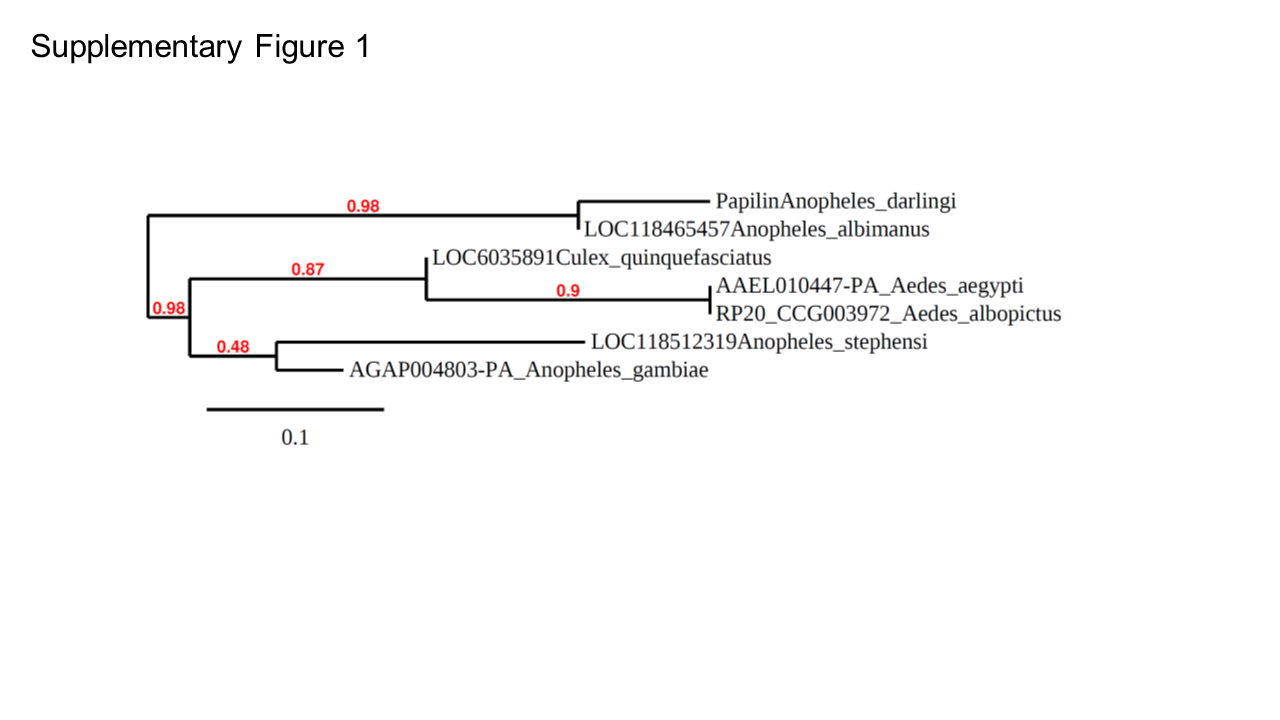

Supplement: FIG S1 [file mbio.03091-21-sf001.tif]

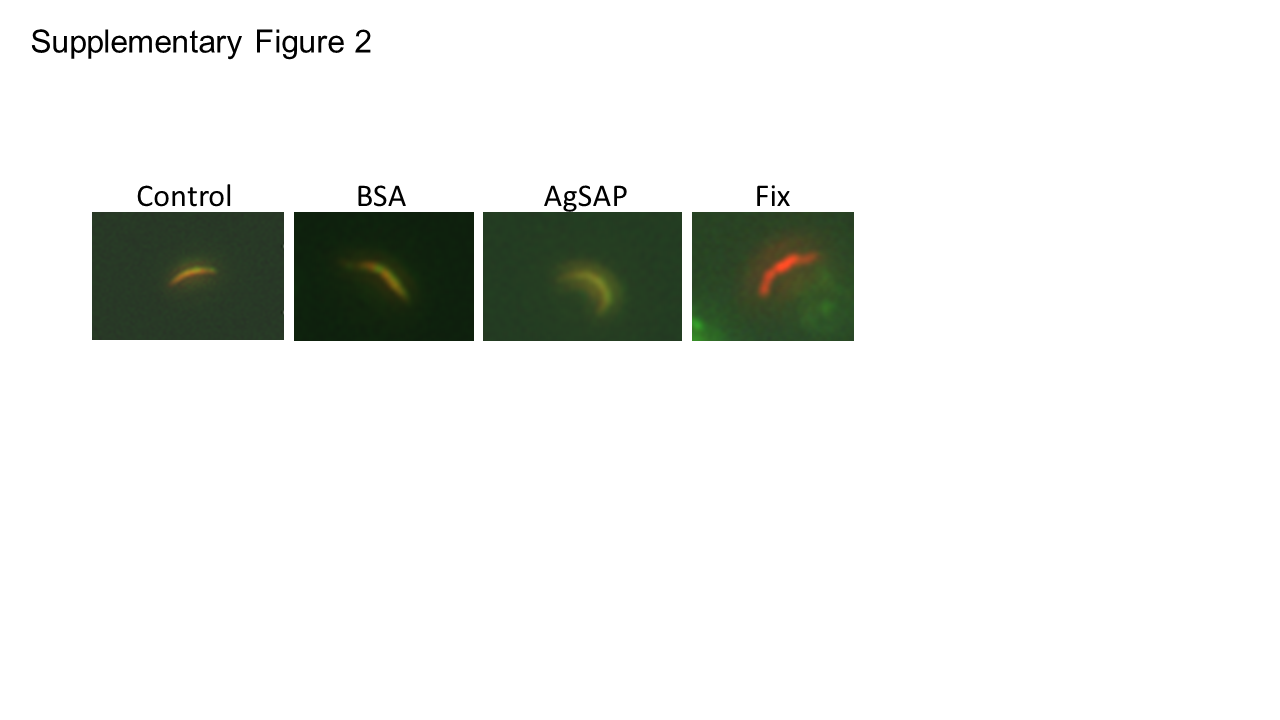

Supplement: FIG S2 [file mbio.03091-21-sf002.tif]

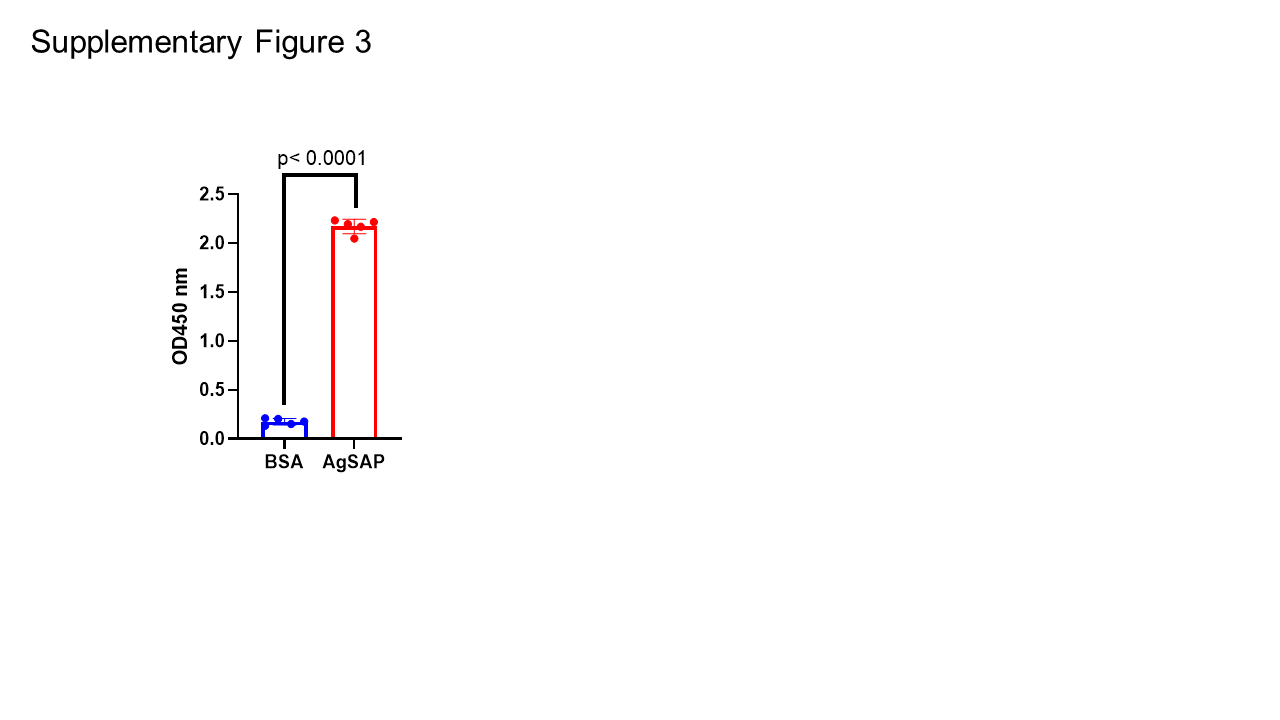

Supplement: FIG S3 [file mbio.03091-21-sf003.tif]

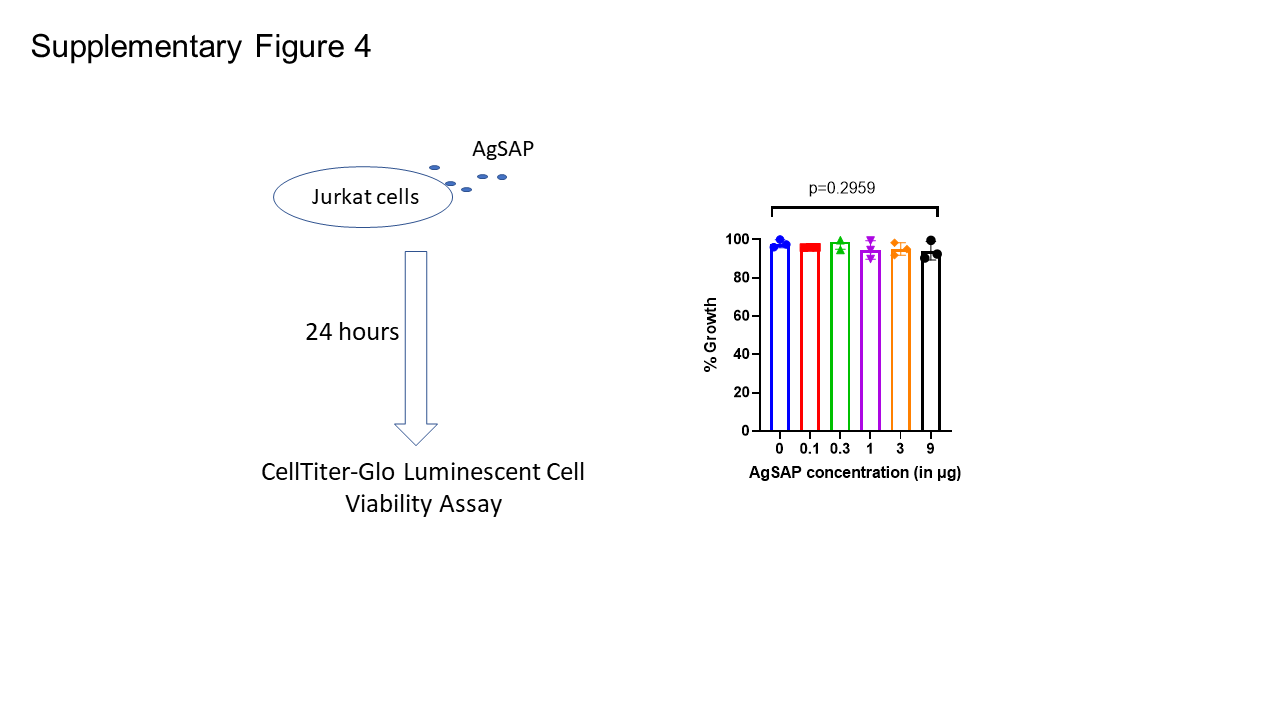

Supplement: FIG S4 [file mbio.03091-21-sf004.tif]

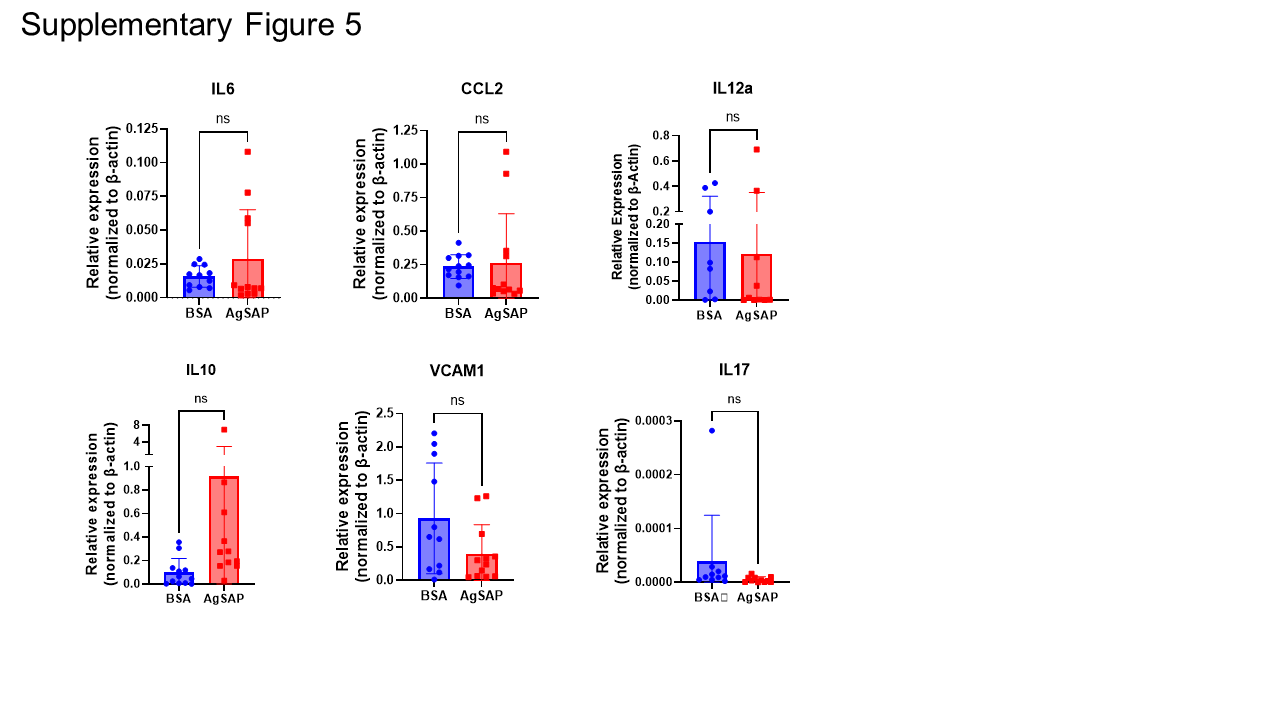

Supplement: FIG S5 [file mbio.03091-21-sf005.tif]
