# Supplementary material for: Immunomodulation by Mosquito Salivary Protein AgSAP Contributes to Early Host Infection by Plasmodium
Source: mBio. 2021 Dec 14;12(6):e03091-21. doi: 10.1128/mBio.03091-21 (PMC8669493; doi:10.1128/mBio.03091-21)
Supplement: TABLE S1 [file mbio.03091-21-st001.docx]

**Supplementary Table 1: List of Oligos used in this study**

**F: Forward Primer, R: Reverse Primer**

| RT AgSAP_F | GGCCGGTTGCGCATGTTTT |
| --- | --- |
| RT AgSAP_R | CGCATTTTCCAGCACTCTA |
| ds AgSAP F | TAATACGACTCACTATAGGGAGCAGAAATATACGTGAGACAAC |
| ds AgSAP F | TAATACGACTCACTATAGGGAGGGAAGAAAATCGGGATCCCC |
| dsLuc F | TAATACGACTCACTATAGGGAGAAACGGATGATGATAACTGGTCCGC |
| dsLuc R | TAATACGACTCACTATAGGGAGAACATCTACTACACTTTCAGCG |
| *P. berghei* 18s F | CCGACTAAGTGTTGGATGAAAA |
| *P. berghei* 18s F | TACTCGCCCCAGAACCCAAAGA |
| Mouse β-actin F | ACGGCCAGGTCATCACTATTG |
| Mouse β-actin R | ACTATGGCCTCAGGGAGTTTTGTCA |
| Mouse HNF4a F | TCAAGGATGAAGAGCTTGCC |
| Mouse HNF4a F | ACGTGTCTGATGTGATCTGC |
| *A. gambiae* β-actin F | GAAGGCTAACCGCGAGAAGATG |
| *A. gambiae* β-actin R | CGCCGGAGTCCAGCACGATA |
| Human GAPDH F | AGGTCGGAGTCAACGGATTTGG |
| Human GAPDH R | GCTCCTGGAAGATGGTGATGGG |
| Human TNFα F | TCCCCAGGGGACCTCTCTCTA |
| Human TNFα R | GAGGGTTTGCTACAACATGGG |
| Mouse TNFα F | AGGCACTCCCCCAAAAGATG |
| Mouse TNFα R | TGGTGGTTTGTGAGTGTGAGG |
| Mouse IL6 F | GAGGATACCACTCCCAACAGACC |
| Mouse IL6 R | AAGTGCATCATCGTTGTTCATACA |
| Mouse IL1b F | CCTTCCAGGATGAGGACATGA |
| Mouse IL1b R | TGAGTCACAGAGGATGGGCTC |
| Mouse IL4 F | ATCATCGGCATTTTGAACGAGGTC |
| Mouse IL4 R | ACCTTGGAAGCCCTACAGACGA |
| Mouse IL10 F | CGGGAAGACAATAACTGCACCC |
| Mouse IL10 R | CGGTTAGCAGTATGTTGTCCAGC |
| Mouse IL12a F | ACGAGAGTTGCCTGGCTACTAG |
| Mouse IL12a R | CCTCATAGATGCTACCAAGGCAC |
| Mouse IL17a F | CAGACTACCTCAACCGTTCCAC |
| Mouse IL17a R | TCCAGCTTTCCCTCCGCATTGA |
| Mouse TGFb F | TGATACGCCTGAGTGGCTGTCT |
| Mouse TGFb R | CACAAGAGCAGTGAGCGCTGAA |
| Mouse CCL2 F | GCTACAAGAGGATCACCAGCAG |
| Mouse CCL2 R | GTCTGGACCCATTCCTTCTTGG |
| Mouse MMP9 F | ATCTCTTCTAGAGACTGGGAAGGAG |
| Mouse MMP9 R | AGCTGATTGACTAAAGTAGCTGGA |
| Mouse ICAM1 F | AAACCAGACCCTGGAACTGCAC |
| Mouse ICAM1 R | AGCTGGAAGATCGAAAGTCCG |
| Mouse VCAM1 F | TGAACCCAAACAGAGGCAGAGT |
| Mouse VCAM1 R | GGTATCCCATCACTTGAGCAGG |
| Mouse IFNγ F | GAGGAACTGGCAAAAGGATGG |
| Mouse IFNγ R | C |
